# Lateralization of Travelling Wave Response in the Hearing Organ of Bushcrickets

**DOI:** 10.1371/journal.pone.0086090

**Published:** 2014-01-21

**Authors:** Arun Palghat Udayashankar, Manfred Kössl, Manuela Nowotny

**Affiliations:** AK Neurobiologie und Biosensorik, Institute for Cell Biology and Neuroscience, Goethe University, Frankfurt/Main, Germany; University of South Florida, United States of America

## Abstract

Travelling waves are the physical basis of frequency discrimination in many vertebrate and invertebrate taxa, including mammals, birds, and some insects. In bushcrickets (Tettigoniidae), the *crista acustica* is the hearing organ that has been shown to use sound-induced travelling waves. Up to now, data on mechanical characteristics of sound-induced travelling waves were only available along the longitudinal (proximal-distal) direction. In this study, we use laser Doppler vibrometry to investigate *in-vivo* radial (anterior-posterior) features of travelling waves in the tropical bushcricket *Mecopoda elongata*. Our results demonstrate that the maximum of sound-induced travelling wave amplitude response is always shifted towards the anterior part of the *crista acustica*. This lateralization of the travelling wave response induces a tilt in the motion of the *crista acustica,* which presumably optimizes sensory transduction by exerting a shear motion on the sensory cilia in this hearing organ.

## Introduction

**Figure 1:**
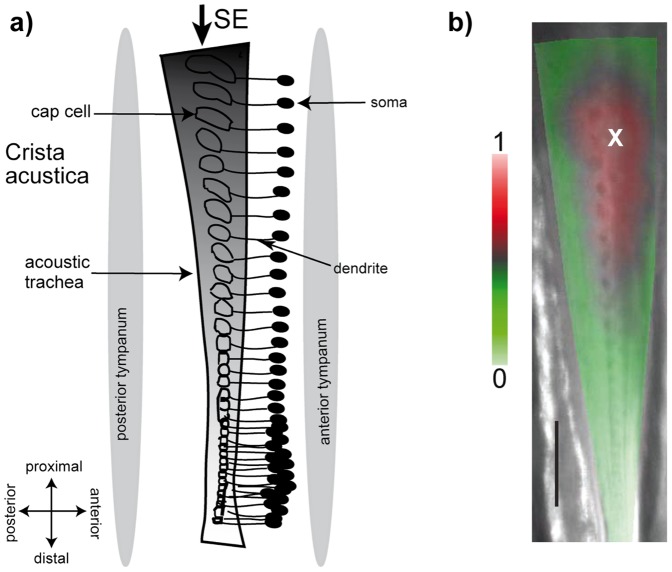
Lateralization of travelling wave amplitude response along the *crista acustica*. (a) Schematic drawing of the *crista acustica*. The *crista acustica* (CA) is lined with a series of cap cells that decrease in size from the proximal to the distal part of the organ. The soma of the sensory neuron is located anterior to the cap cell and is connected to it via a dendrite. The CA is slightly curved towards the anterior side, i.e., it has a convex shape towards the dendrites. (b) Color-coded 2D velocity response of the CA superimposed on an image of the preparation for a right leg stimulated with 9 kHz and 80 dB SPL are shown. The cross indicates the maximum velocity response. Abbreviation: SE  =  sound entry.

Bushcrickets (Tettigoniidae) perceive a wide frequency bandwidth of substrate- and air-borne sounds ranging up to ultrasonic frequencies. The hearing organ of bushcrickets, the *crista acustica* (CA; Fig. 1a), is located in each leg of the animal. In the forelegs of the bushcricket, the CA processes high-frequency sound between ∼5 and at least 80 kHz [1]–[2]. The primary entry for high-frequency sound to the CA can be found in the prothoracic region of the animal, which is an oval-shaped opening known as the spiracle [3]–[5]. The spiracle channels sound into the adjoining horn-shaped acoustic trachea (AT), where acoustic sound is amplified [6]. The CA is located on top of the AT in the tibial region of the forelegs. Sensory cells of the CA detect motion of the air-filled AT through a mechano-electrical transduction process. An alternative sound entry path is via the tympana. However, the response of the CA to stimulation by this pathway is restricted to low frequencies [7]. The tympana are compliant plate-like structures in the forelegs, bordering the AT on the anterior and posterior sides.

The CA of *Mecopoda elongata,* the bushcricket species investigated here, consists of about 45 auditory sensillae, arranged along the longitudinal axis (proximal-distal) of the hearing organ (Fig. 1a). Each sensillum is composed of a sensory neuron and supporting cells. The transduction apparatus is placed at the tip of the sensory neuron’s dendrite [8], which is surrounded by a so-called scolopale cell and covered by a cap cell. Somata of the sensory neurons are placed on the anterior side of the CA (Fig. 1a).

Using wavelength and wave velocity data, we have shown recently that longitudinal characteristics of sound-induced travelling waves in the CA are comparable to those found in mammals [9]. However, in mammals, very little experimental data [10]–[13] are available on the radial (anterior-posterior direction) structure of travelling waves due to difficulties associated with the location and shape of the mammalian cochlea for experimentation. In contrast, the CA offers the opportunity to more easily assess the radial properties of travelling waves by using a combination of a scanning laser Doppler vibrometer and an *in-vivo* dorsal preparation of the CA.

## Materials and Methods

In this study, we used 13 bushcrickets (*Mecopoda elongata,* Orthoptera; Tettigoniidae Phaneropterinae) from our own breeding colony. The animals were anesthetized with CO_2_ before the hind-legs, the mid-legs, and the cuticle of the foreleg were removed to expose the CA. Vibration responses to pure-tone stimulation under free field conditions were measured with a laser Doppler vibrometer. A more detailed description of the experimental procedure can be found elsewhere [4]. We used a high spatial resolution of ca. 10 µm for the measurement points along the radial direction of the mesh grid in the region corresponding to the maximum displacement of the longitudinal travelling wave (Fig. S1).

### (a) Quantification of volume response and lateralization

X, Y coordinates and maximum values of velocity from any point on the measured grid were exported to MATLAB (Matworks Inc., Nauticks, USA) after low frequency noise (velocity <10 µm/s) was filtered out. To standardize the data, the data points were first projected onto a tonotopy-based mesh grid and later interpolated using Delaunay triangulation including the application of a median filter to reduce noise introduced by interpolation. We used the volumetric response to quantify lateralization of the travelling wave response with respect to the scolopidial axis (Fig. S2A). The scolopidial axis was defined as a proximal-distal line joining two points (X1, Y1) and (X2, Y2) as shown in Fig. S2B. The coordinates (X1, Y1) and (X2 and Y2) correspond to the center of the scolopidia lying above and below the best frequency (BF) location. The scolopidial axis divides the CA into an anterior and posterior surface.

Volumetric response was quantified by dividing the uniform interpolated measurement grid into triangles. The mean height (velocity measurement) of each Delaunay triangle was estimated by averaging the velocity data at its vertices. The volume beneath each triangle was obtained by multiplying the area of the triangle with the corresponding mean height. The volumes were then summed to get the volume of the entire surface using the following equation:
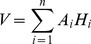
(1)


where V is the volume, A*i* is the area of the Delaunay triangle and H*i* is the corresponding height of the triangle. In order to quantify lateralization, a lateralization index (LI) was defined according to the following equation:
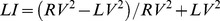
(2)


where LV is the volume to the left of the scolopidial axis and RV is the volume to the right of the scolopidial axis. Depending on whether it was the right or left leg, the anterior surface of the CA was on the right or left side with respect to the scolopidial axis. An example is shown in Fig. S2.

### (b) Quantification of tilt

In order to quantify the tilt we exported the radial displacement profile measured along the breadth of the organ at the BF location. The slope at the scolopidia center (X0) was determined with the following equation:
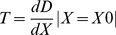
(3)


where D represents displacement and X represents distance.

## Results

In the present study, we measured and characterized the properties of travelling waves along the radial direction with high spatial resolution at the region with the highest proximodistal velocity response to a given stimulus frequency.

### (a) Maximum amplitude of the travelling wave is lateralized

**Figure 2:**
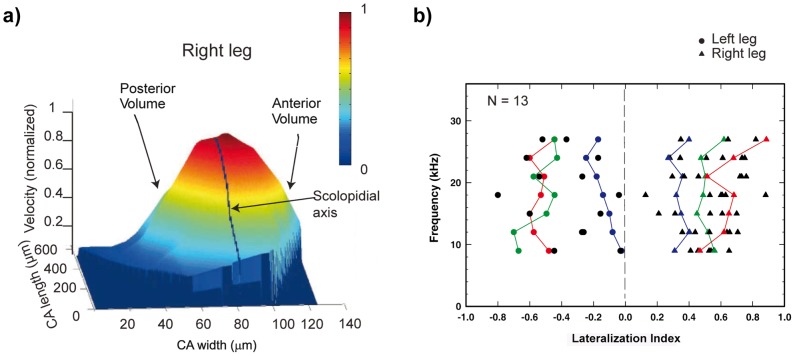
Quantification of amplitude lateralization of travelling wave. (a) Normalized volumetric response of the CA for a single preparation is displayed. The scolopidia axis, marked by a black line, is used to divide the area into anterior and posterior volume response sides. (b) Values of LIs for 13 different animals show a clear segregation of left and right legs. Representative examples are marked with colored lines (green, red, blue).

When the scolopidial axis was taken as the midline, the analysis of the radial travelling wave structure revealed that the maximum response was always shifted towards the anterior part of the *crista acustica* (Fig. 1b). In order to quantify this maximum shift (lateralization) for the measured range of frequencies, the volumetric response on each side of the scolopidia axis was calculated (Fig. 2a). The resulting lateralization index (LI) describes the ratio of the difference between the two sides in relation to the entire volumetric response. For the left legs (N  =  5), negative values of LI indicate an anterior lateralization and positive values indicate a posterior lateralization and *vice versa* for the right legs (N  =  8). After the investigation of 13 different preparations, it became obvious that for all left legs the values are negative and for all right legs all the values are positive (Fig. 2b). This quantification implies that regardless of the actual applied frequency, lateralization of the travelling wave energy along the CA is always anterior relative to the scolopidial axis. An estimation of the LI for stimulation frequencies above 30 kHz was not possible because the scan resolution tended to approach the width of the trachea, making a clear quantification of lateralization impracticable.

### (b) Lateralization causes a tilt of the CA

**Figure 3:**
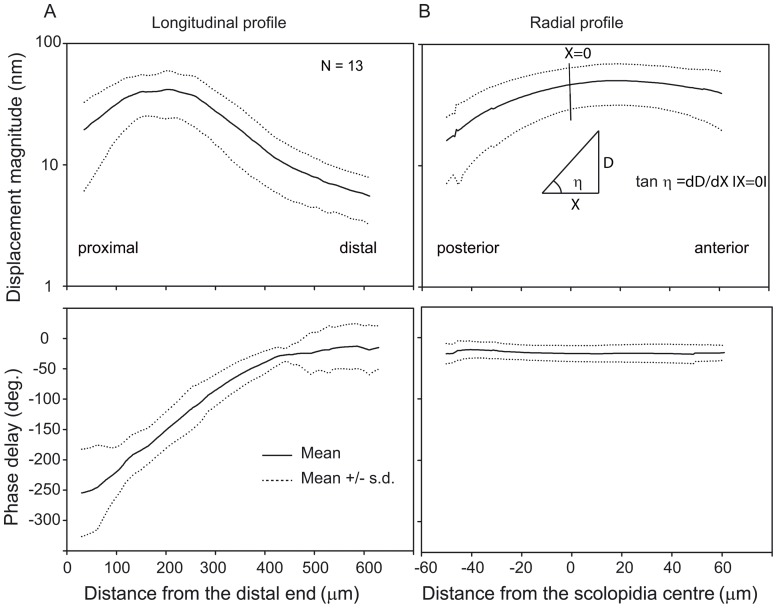
Magnitude and phase response of the *crista acustica*. The magnitude (upper panels) und phase (lower panels) responses of the travelling wave for the longitudinal (a) and radial (b) direction at 9 kHz and 80 dB SPL from 13 different animals are shown. The longitudinal response profiles were obtained along the scolopidial axis (proximal-distal), while the radial response profiles were from the location of maximum velocity response and were measured along the anterior-posterior direction. The vertical line in the upper panel of b indicates approximate location of the scolopidia. The solid lines represent the mean and the dotted lines represent ± standard deviation. The reported tilt is a measure of the slope (tan η) at the scolopidia location (0 µm).

Magnitude and phase response are used to characterize the properties of the travelling waves in the longitudinal and radial direction along the CA. In a tonotopic manner, the longitudinal profiles of magnitude displacement of the sound-induced travelling waves have a frequency-dependent maximum distribution along the CA. For a stimulation with 9 kHz and 80 dB SPL, the maximum displacement is reached at a BF location of ∼200 µm (Fig. 3a, upper panel). The longitudinal phase response of the travelling waves exhibits a progressive delay from the distal end of the CA towards the BF location (Fig. 3a, lower panel). Measurements of the radial profiles at the BF location show a gradient in the displacement amplitude at the scolopidia location, indicating that the lateralization of the travelling wave response causes a tilt of the CA (Fig. 3b, upper panel). The scolopidia (position marked by a vertical line in Fig. 3b, upper panel) are localized on the posterior slope of the mechanical response. This slope was always positive with a mean of 0.45×10^−3^ (± 0.18×10^−3^) across the thirteen preparations. No significant change in phase response was seen along the radial direction (Fig. 3b, lower panel).

## Discussion

Using the easily accessible bushcricket hearing organ as a model system, we studied the radial and longitudinal structure of travelling waves. Our results reveal that the magnitude response of the CA is lateralized in relation to the scolopidial axis. This lateralization of the response causes a tilt in the magnitude response of the CA. Presumably this tilt of the CA induces a shear motion on the cilia of the sensory neuron, which transmits the force to the mechano-sensitive transduction channel to open it. In order to convey the force to the transduction channel, we expect the channels to be located posteriorly, on the cilium, where stretching of the membrane would be maximal.

Mathematical models predict that lateralization of travelling wave energy occurs in the mammalian cochlea and is caused by its curvature [14]. This lateralization of travelling wave energy presumably has a functional significance. It leads to a dynamic tilt of the basilar membrane as the wave progresses through the cochlea [14]–[15] leading to a shear motion of the hair-cell bundles. In the present study, we show that, in the hearing organ of bushcrickets, the traveling wave response is lateralized. This leads to a tilt of the CA in response to acoustic stimulation as predicted for the mammalian organ of Corti.

Since lateralization in the mammalian cochlea is thought to be a result of its curvature [14]–[15], we quantified the curvature of the CA. Quantification of the radius of curvature of CA, however, did not lead to a conclusive inverse relationship with the lateralization index. This could be due to the following reasons. The lateralization index cannot be used as a measure of traveling wave energy as we did not calibrate our measurement setup to derive pressure from velocity, hence we do not have a measure of the potential energy of the system. Further, since kinetic energy (KE) has a mass term in it (KE = m*v^2^), a static mass gradient along the posterior-anterior axis (radial direction) could also lead to lateralization. This is likely to be the case as our mechanical measurements of the CA motion covered the anterior spread of the dendrites and the soma of the neurons. Further investigation of the material properties of the structures in the CA combined with a mechanical model may help us to better understand the source of the observed lateralization in bushcrickets.

## Supporting Information

Figure S1 **Tonotopy-based measurement grids for determining radial structure of traveling waves.** Tonotopy identified along the CA from longitudinal traveling waves was used to optimize scan time. Measurements grids shown here were used to determine the radial structure of travelling waves in the CA for different frequencies. The figure shows examples of measurement grids for 6 (A), 12 (B), 21 (C), and 30 kHz (D) in a representative preparation. Scale bar: 100 µm.
(TIF)

Figure S2 **Lateralization of the displacement response of the CA.** (A) Top view of the scan points (square dots) superimposed on a typical preparation of the CA. The dashed line in the center represents the scolopidial axis (SA). (B) Top view of the interpolated surface corresponding to a measurement using a stimulus frequency of 21 kHz at 80 dB SPL. The two points (X1, Y1) and (X2, Y2) on the scolopidia proximal and distal to the BF location (BF loc.) were used to determine the scolopidial axis (SA). (C) Perspective view of the normalized velocity magnitude response is shown. The cut at the center indicates the location of the scolopidial axis (SA). The volume of the maximum amplitude profile was calculated anterior and posterior to the SA. The formula used to calculate lateralization index (LI) is indicated in the figure. Scale bar (A): 100 µm.
(TIF)
