# Supplementary figures and images for: Lateralization of Travelling Wave Response in the Hearing Organ of Bushcrickets
Source: PLoS One. 2014 Jan 21;9(1):e86090. doi: 10.1371/journal.pone.0086090 (PMC3897617; doi:10.1371/journal.pone.0086090)

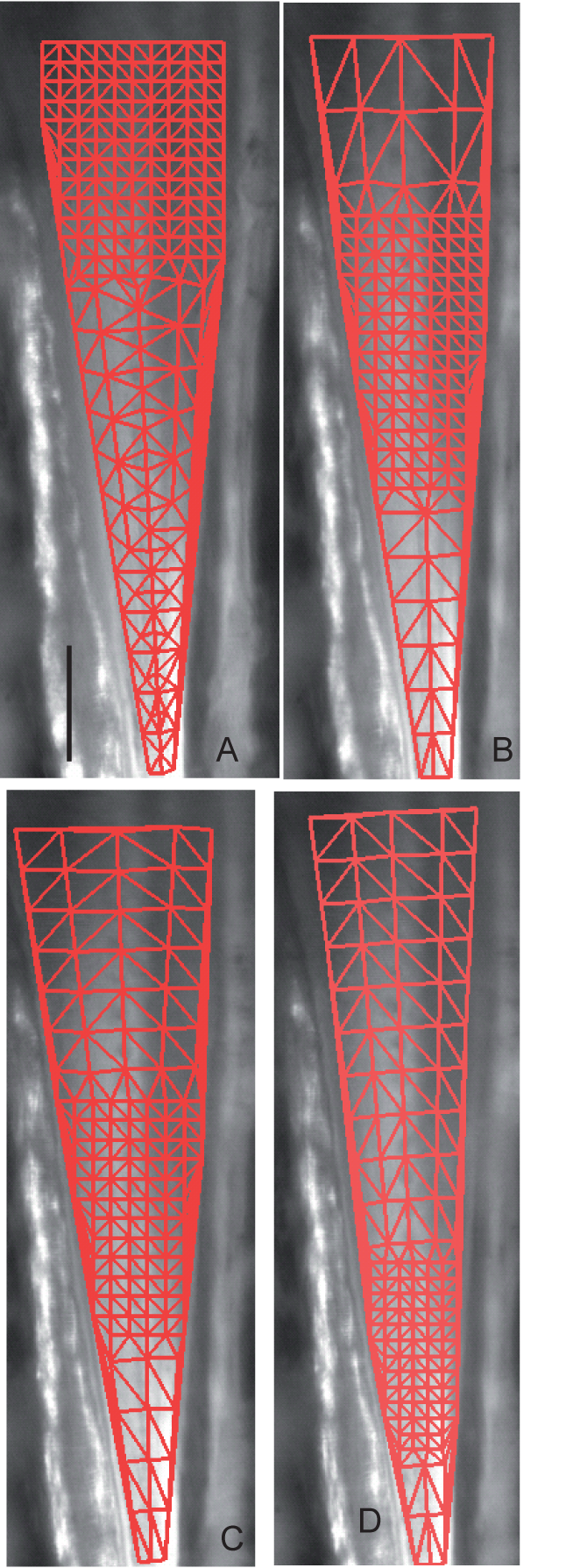

Supplement: Figure S1 — Tonotopy-based measurement grids for determining radial structure of traveling waves. Tonotopy identified along the CA from longitudinal traveling waves was used to optimize scan time. Measurements grids shown here were used to determine the radial structure of travelling waves in the CA for different frequencies. The figure shows examples of measurement grids for 6 (A), 12 (B), 21 (C), and 30 kHz (D) in a representative preparation. Scale bar: 100 µm. (TIF) [file pone.0086090.s001.tif]

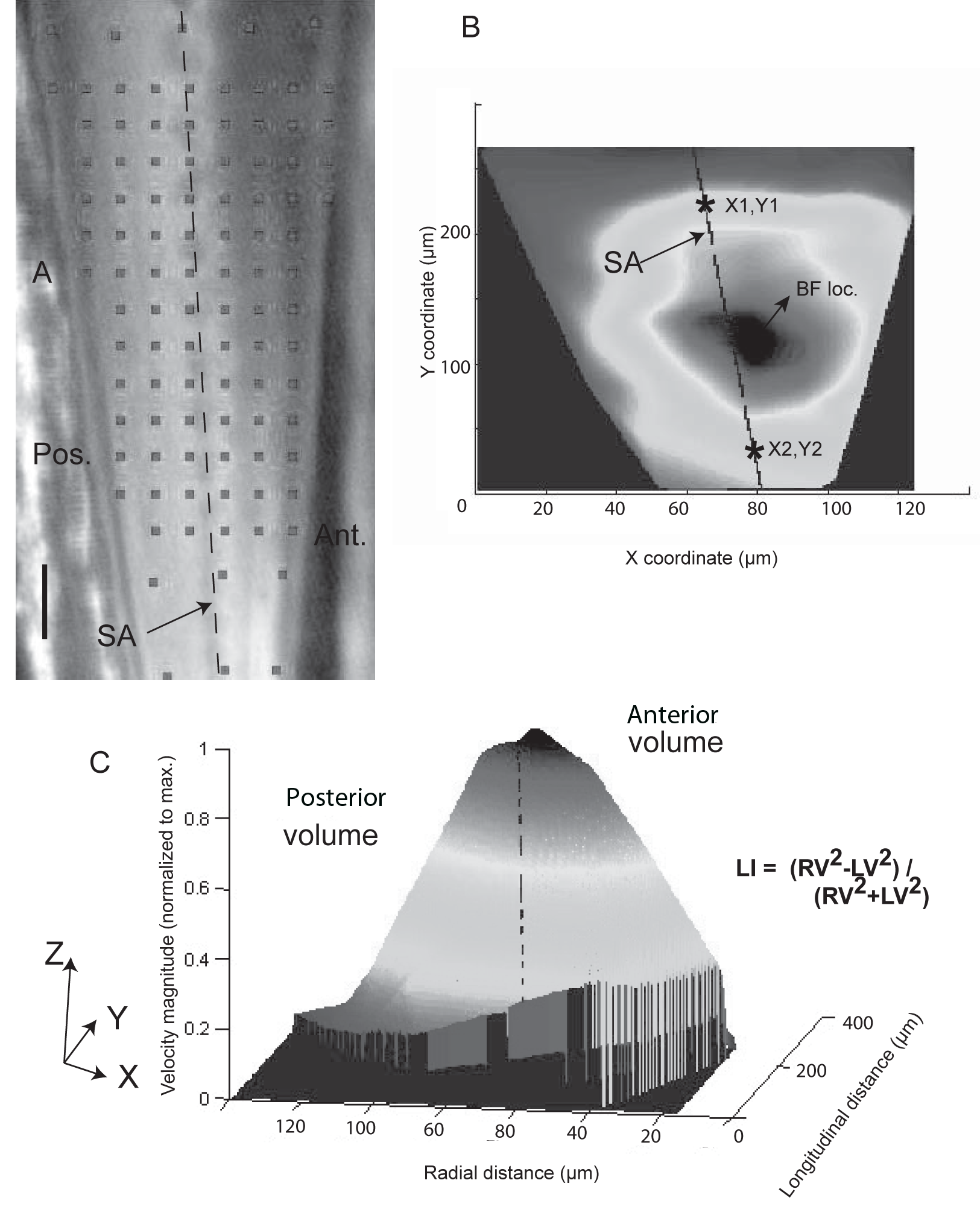

Supplement: Figure S2 — Lateralization of the displacement response of the CA. (A) Top view of the scan points (square dots) superimposed on a typical preparation of the CA. The dashed line in the center represents the scolopidial axis (SA). (B) Top view of the interpolated surface corresponding to a measurement using a stimulus frequency of 21 kHz at 80 dB SPL. The two points (X1, Y1) and (X2, Y2) on the scolopidia proximal and distal to the BF location (BF loc.) were used to determine the scolopidial axis (SA). (C) Perspective view of the normalized velocity magnitude response is shown. The cut at the center indicates the location of the scolopidial axis (SA). The volume of the maximum amplitude profile was calculated anterior and posterior to the SA. The formula used to calculate lateralization index (LI) is indicated in the figure. Scale bar (A): 100 µm. (TIF) [file pone.0086090.s002.tif]
